# Why public health cannot be led by doctors only

**DOI:** 10.1097/JS9.0000000000000030

**Published:** 2023-03-24

**Authors:** Fatima Yasin, Qasim Mehmood, Hassan Mumtaz

**Affiliations:** a King Edward Medical University, Lahore, Pakistan; b Health Services Academy, Islamabad

It has been always a point of debate when it comes to leadership of public health. However, there has always been a competition between doctors and nondoctors to gain this leadership in their hands. Public health has always been an important task as it encompasses the life of every person in society and is of prime importance in maintaining the quality of life. Moreover, public health leaders are responsible for identifying national health problems and formulating policies to cater to them. They are also responsible for collaborating with international stakeholders to solve public health problems as recently has been seen in the coronavirus disease-2012 pandemic^1^. Public health deals with the promotion, maintenance as well protection of the health of individuals. Such multidisciplinary work requires a team effort. 4 As are used to encompass these multidimensional activities of public health: academics, activism, administration, and advocacy^2^.

There has always been a notion that medical and public health are the same. A person who is able to treat one person is ready to serve the duty of serving the whole nation too. However, both fields work on different ways of analysis. Medical health deals with the analysis of the health of a person while public health deals with the statistical analysis of the overall prevalence and incidence of a health problem in a community. For example, a booster for coronavirus disease-2012 will protect the health of an individual but what is the effect of a booster on the health of the community comes under public health. Will it be able to achieve herd immunity to protect the whole community will be the topic of discussion under public health^3^. A study from Bangladesh also showed that both the roles can be served well by doctors at a bigger level but at the periphery, but at the periphery, both the roles get amalgamated and the distinction becomes unclear.^4^

Historically, it was led by doctors as they were the ones to identify any medical health problem. Then as time passes, Rudolph Virchow, a German medical doctor, came forward and coined the term “social medicine”^3^. There are some core fields of public health such as medical, biological, behavioral as well as environmental beside social medicine. So advocates of one particular field cannot exclusively be the charge bearers of the others too because public health is achieved by the interplay of all these core elements.

The prevention paradox theory of Geoffrey Rose fits here well as medical leaders will be directed in making strategies that will be aiming at secondary or tertiary prevention of the diseases. Their focus will be on the ill or high-risk patients and this will lead to a bias in public health. In this way, primary prevention of the disease will be neglected and the motto of public health will be unable to be achieved. Medical leaders have been deficient in other core disciplines and have been influenced by downplaying the roles of other core disciplines^5^. They are lacking an understanding of the other disciplines and have been unable to manage it all by themselves till now. Moreover, leadership skills are a weak point in many medical persons as they have not been taught in an effective manner during their course of study. Despite the fact that it has always been considered a core competence in medical institutions, there has been no reinforcement to build this across the whole curriculum^6^.

Public health has suffered a lot because it has been divided and dispersed among the power rule by one dominant discipline. The interplay of the core disciplines should be achieved in a balanced way so that there is a well-managed and more unbiased public health work. A knowledgeable enthusiast and a genuine leader should be the charge bearer of public health, whether from medical background or not. Moreover, the medical curriculum should be devised in such a way that leadership skills are taught in an effective way as it will lead them to be better public health officials^6^. Doctors should have a population approach to the clinical practice to bridge the gap they are facing while leading the high offices in public health^5^. Public health should be trained with the new innovative IT as well as leadership competency models so that they are competent enough to deal with the complexities of health problems of the public. Public health capacity should also be built and enhanced in such a way that we can be able to reduce the inequalities in public health. Moreover, public health degrees are traditional are not generate enough leadership skills. Therefore, these courses should also be revised to the extent that the public health degree holders are able to compete for the higher posts and are able to deal with the conflicts of public health^3^.

There are four phases of leadership development when it comes to making an individual an effective leader. The first step is the initiation of starting this journey, and second, identification of such a degree and getting a public health degree too. The third step is the development by experience and working under such circumstances and fourth is the expansion of his capacities and abilities to serve the cause^7^.

## Ethical approval

None.

## Sources of funding

None.

## Authors' contribution

H.M.: concept. F.Y.: manuscript writing. Q.M.: editing and review.

## Conflicts of interest disclosure

The authors declare that they have no financial conflict of interest with regard to the content of this report.

## Research registration unique identifying number (UIN)

None.

## Guarantor

Hassan Mumtaz.

## Provenance and peer review

Not commissioned, externally peer-reviewed.

## Data statement

Data will be made available upon request.
